# Assessment of clinical deterioration and progressive organ failure in moderate-severity emergency department sepsis patients

**DOI:** 10.1186/cc11788

**Published:** 2012-11-14

**Authors:** R Arnold, L Glaspey, S Hollenberg, S Trzeciak

**Affiliations:** 1 Cooper University Hospital, Camden, NJ, USA

## Background

The PRE-SHOCK population, ED sepsis patients with tissue hypoperfusion (lactate of 2.0 to 3.9 mM), commonly deteriorates after admission and requires transfer to critical care. The objective was to determine the physiologic parameters and disease severity indices in the ED PRE-SHOCK sepsis population that predict clinical deterioration. We hypothesized that neither initial physiologic parameters nor organ function scores will be predictive.

## Methods

A retrospective analysis of a prospectively maintained registry of sepsis patients with lactate measurements in an urban, academic medical center. The PRE-SHOCK population was defined as adult ED sepsis patients with either elevated lactate (2.0 to 3.9 mM) or transient hypotension (any sBP <90 mmHg) receiving intravenous antibiotics and admitted to a medical floor. Consecutive patients meeting PRE-SHOCK criteria were enrolled over a 1-year period. Patients with overt shock in the ED, pregnancy, or acute trauma were excluded. The primary patient-centered outcome was increased organ failure (Sequential Organ Failure Assessment (SOFA) score increase >1 point, mechanical ventilation or vasopressor utilization) within 72 hours of admission or in-hospital mortality.

## Results

We identified 248 PRE-SHOCK patients from 2,649 screened. The primary outcome was met in 54% of the cohort and 44% were transferred to the ICU from a medical floor. Patients meeting the outcome of increased organ failure had a greater shock index (1.02 vs. 0.93, *P *= 0.042) and HR (115 vs. 105, *P *< 0.001) with no difference in initial lactate, age, MAP or exposure to hypotension (sBP <100 mmHg). There was no difference in the Predisposition, Infection, Response, and Organ dysfunction (PIRO) score between groups (6.4 vs. 5.7, *P *= 0.052). Outcome patients had similar initial levels of organ dysfunction but had higher SOFA scores at 24, 48, and 72 hours, a higher ICU transfer rate (60 vs. 24%, *P *< 0.001) and increased ICU and hospital lengths of stay.

## Conclusion

The PRE-SHOCK sepsis population has a high incidence of clinical deterioration, progressive organ failure, and ICU transfer. Physiologic data in the ED were unable to differentiate the PRE-SHOCK sepsis patients who developed increased organ failure. This study supports the need for an objective organ failure assessment in the emergency department to supplement clinical decision-making.

